# Age-specific HPV genotype distribution and associations with cervical lesions and vaginal microecological status in Northeast China: a retrospective study of 73,828 individuals

**DOI:** 10.3389/fcimb.2026.1993128

**Published:** 2026-10-30

**Authors:** Yulu He, Yijia Pan, Zhijue Sheng, Yong Liu, Sijia Hao, Weihua Tong, Yanfang Jiang

**Affiliations:** 1 Genetic Diagnosis Center, The First Hospital of Jilin University, Changchun, China; 2 Pharmaceutical Sciences, School of Pharmaceutical Sciences, Jilin University, Changchun, China; 3 Center of Obstetrics and Gynecology, The First Hospital of Jilin University, Changchun, China

**Keywords:** epidemiology, human papillomavirus, invasive cervical cancer, type distribution, vaginal microecological imbalance

## Abstract

**Background:**

Persistent high-risk human papillomavirus (HR-HPV) infection is a major etiological factor in cervical carcinogenesis. HPV prevalence and genotype distribution show substantial geographic heterogeneity across China, highlighting the need for region-specific epidemiological data. This study characterized age-specific HPV infection and genotype patterns in Northeast China and further examined their associations with cervical pathology and vaginal microecological status.

**Methods:**

We retrospectively analyzed HPV genotyping data from 73,828 individuals tested between 2020 and 2024. Histopathological data were available for 8,755 women, and vaginal microecological assessments were available for 2,428 women. Age-related differences, HPV genotype distributions, infection patterns, and their associations with cervical pathology and vaginal microecological categories were evaluated using nonparametric and χ² tests.

**Results:**

Overall HPV positivity was 20.83% (15,376/73,828) and showed a bimodal age distribution, with the highest prevalence among participants aged < 25 years (34.44%) and a second peak at 60–65 years (25.90%). HPV16, HPV52, and HPV58 were the three most frequently detected genotypes. Single HR-HPV infection differed significantly across cervical histopathological categories and was most frequent in cervical cancer (57.81%, *P* < 0.001). HPV positivity also differed across vaginal microecological categories (*P* < 0.001), with the highest rate observed in the bacterial vaginosis group (32.66%); however, no individual HPV genotype remained significantly associated with microecological category after FDR correction.

**Conclusion:**

These findings provide regional epidemiological evidence for HPV surveillance and cervical cancer prevention planning and support further prospective investigation of the relationship between vaginal microecological alterations and HPV infection.

## 1 Introduction

Cervical cancer remains a major global health burden, with approximately 661,000 new cases and 348,000 deaths estimated worldwide in 2022. China accounted for approximately 151,000 new cases and 56,000 deaths during the same year (Bray et al., 2024). Persistent infection with high-risk human papillomavirus (HR-HPV) is the principal etiological factor underlying cervical precancerous lesions and cervical cancer (Schiffman et al., 2007). Oncogenic HPV infection can disrupt epithelial cell-cycle regulation and contribute to malignant transformation, with HPV16 and HPV18 together accounting for approximately 70% of cervical cancers worldwide Wei et al., 2024). Other HR-HPV genotypes also contribute to cervical and other anogenital malignancies, whereas low-risk HPV types are predominantly associated with benign anogenital lesions (Chaturvedi et al., 2011; Jemal et al., 2013).HPV infection is also clinically relevant in men. Low-risk HPV genotypes, particularly HPV-6 and HPV-11, are commonly associated with genital warts, whereas persistent infection with certain high-risk HPV genotypes has been linked to penile, anal, and oropharyngeal cancers. Characterizing the distribution of HPV genotypes in men may provide complementary epidemiological information on HPV-associated diseases beyond cervical cancer.

Prophylactic HPV vaccination and HPV-based cervical screening are central components of cervical cancer prevention (Wirtz et al., 2022; Melnikow et al., 2018). However, HPV epidemiology remains heterogeneous across populations, with considerable geographic variation in both overall prevalence and genotype distribution. Recent nationwide data from China further demonstrate substantial regional differences in HPV prevalence and confirm HPV52, HPV58, and HPV16 as major circulating high-risk genotypes, although their relative predominance varies across populations (Han et al., 2025; Yan et al., 2026; Gao et al., 2026). Regional studies from Shanghai, Wuhan, Chengdu, and Guangzhou similarly report distinct genotype profiles, further underscoring geographic heterogeneity within China (Cheng et al., 2025; D. Wang et al., 2024; J. Zhang et al., 2024; Huiyun et al., 2023). Age is another important epidemiological determinant, with recent Chinese studies consistently showing elevated HPV prevalence among younger women and a second increase in older age groups. Characterizing these regional and age-specific patterns is therefore important for defining the local burden of HPV infection and informing regionally appropriate screening and prevention strategies.

Beyond viral genotype and host age, the cervicovaginal microenvironment has emerged as an important clinical correlate of HPV infection and cervical disease (Houvessou et al., 2026). *Lactobacillus*-dominant vaginal communities, particularly those enriched in *Lactobacillus crispatus*, are generally associated with lower vaginal pH and a more stable mucosal environment, whereas *Lactobacillus*-depleted states characterized by greater microbial diversity and enrichment of anaerobic bacteria have been reported more frequently in women with HPV infection or cervical abnormalities (Žukienė et al., 2025; Bautista et al., 2026). Recent systematic reviews and longitudinal studies suggest that stable *Lactobacillus*-dominated community states are more frequently observed in women who subsequently clear HPV or experience lesion regression, whereas *Lactobacillus*-depleted or anaerobe-enriched states are more often associated with HPV persistence and unfavorable cervical lesion outcomes; however, substantial heterogeneity across studies precludes firm causal inference (Shi et al., 2022; Houvessou et al., 2026).Recent studies have examined associations between individual vaginal infections and HPV genotypes (J. Wang et al., 2026), but HPV epidemiology, histopathological severity, and vaginal microecological status are frequently investigated as separate clinical dimensions. A large-scale assessment integrating these features may therefore provide a more comprehensive description of HPV infection patterns and their clinical context.

Accordingly, we retrospectively analyzed HPV genotyping data from 73,828 individuals tested in Northeast China between January 2020 and December 2024. We characterized age-specific HPV prevalence, genotype distribution, and patterns of single and multiple infection, and further examined their associations with cervical histopathological categories and vaginal microecological status in available clinical subsets. By integrating large-scale HPV epidemiological data with pathological and vaginal microecological information, this study aimed to provide a comprehensive regional profile of HPV infection and to identify clinically relevant patterns that may inform HPV surveillance and cervical cancer prevention.

## 2 Materials and methods

### 2.1 Study population

This retrospective study included 73,828 unique individuals (69,771 females and 4,057 males) who underwent HPV genotyping at the Gene Diagnosis Center of the First Hospital of Jilin University between January 2020 and December 2024. For individuals with repeated HPV tests during the study period, only the first test was retained as the index HPV test. Exclusion criteria included pregnancy, prior hysterectomy, documented HIV infection, active systemic infection, and autoimmune disease.

Additional clinical data were obtained by retrospectively matching HPV genotyping records with available medical records. Cervical histopathological diagnoses were available for 8,755 women, who were included in the histopathological analysis based on the availability of both HPV testing and cervical pathology records. Vaginal microecological assessments were available for 2,428 women with corresponding HPV genotyping results. When multiple histopathological or microecological records were available, the examination closest to the index HPV test was selected.

For the wart-related analysis, HPV genotyping records were matched with clinical diagnoses of genital or common warts. This identified 734 participants with documented wart-related diagnoses, including 256 males and 478 females. As systematic assessment for HPV-associated lesions was not available for all male participants, the wart-related analysis was restricted to individuals with recorded clinical diagnoses. Male participants were included in the overall epidemiological and wart-related analyses but not in the cervical histopathological or vaginal microecological analyses.

The study protocol was approved by the Ethics Committee of the First Hospital of Jilin University (approval no. 22K045-001). Because this was a retrospective study based on routinely collected clinical data, the requirement for informed consent was waived by the Ethics Committee. All data were analyzed in accordance with relevant institutional and ethical requirements.

### 2.2 Grouping criteria

Participants were stratified into 10 age groups to characterize age-specific HPV infection patterns: <25, 25–29, 30–34, 35–39, 40–44, 45–49, 50–54, 55–59, 60–65, and >65 years.

Cervical histopathological diagnoses were classified according to the 2020 WHO Classification of Female Genital Tumours (Höhn et al., 2021) and the Chinese Guidelines for Cervical Cancer Diagnosis and Treatment (China, 2022). Histopathological findings were categorized as chronic cervicitis, low-grade squamous intraepithelial lesion (LSIL; corresponding predominantly to cervical intraepithelial neoplasia grade 1 [CIN1]), high-grade squamous intraepithelial lesion (HSIL; including CIN2 and CIN3). Cervical cancer included squamous cell carcinoma, adenocarcinoma, and adenosquamous carcinoma. Cervical lesion categories were defined according to histopathological diagnoses rather than cervical cytological findings.

### 2.3 Sample collection and storage

For female participants, excess cervical secretions were gently removed before sampling. Cervical cells were collected by rotating a cytobrush 3–5 times at the squamocolumnar junction and transferred into the designated preservation medium (Hybribio Limited, Chaozhou, China).

For male participants, genital scraped cells were collected by physicians using sterile nylon-flocked swabs. The sampling sites included the urethral epithelium, anus, or penile epidermis, depending on clinical indications. Specifically, for urethral sampling, the swab was gently inserted approximately 2–4 cm into the urethral orifice and rotated for 5–10 seconds to scrape mucosal exfoliated cells. If visible warts or ulcers were present, the lesion surface was repeatedly swabbed. For coronal sulcus/glans sampling, the swab was rotated and rubbed against the coronal sulcus, glans, or inner prepuce to ensure adequate cell collection. For perianal/anal sampling, the swab was inserted approximately 3–5 cm into the anal canal and rotated, or scraped at the junction of the perianal skin and anus.

All Samples are stored at 4 °C, and DNA extraction was performed according to the manufacturer’s instructions.

### 2.4 HPV genotyping

HPV detection utilized a PCR-based reverse dot blot hybridization kit (Hybribio Limited) targeting 14 high-risk types (16, 18, 31, 33, 35, 39, 45, 51, 52, 56, 58, 59, 66, 68), one intermediate-risk type (53), and six low-risk types (6, 11, 42, 43, 44, 81). The procedure involved DNA extraction per the manufacturer’s protocol, PCR amplification (initial denaturation at 95 °C for 9 min; 40 cycles of 95 °C for 20 s, 55 °C for 30 s, and 72 °C for 30 s; final extension at 72 °C for 5 min), and genotyping via hybridization with type-specific probes immobilized on nylon membranes. Negative controls (nuclease-free water) and positive controls were included in all assays.

### 2.5 Histopathological assessment

Histopathological diagnoses were established by qualified pathologists and classified according to the 2020 WHO Classification of Female Genital Tumours and the relevant Chinese clinical guidelines. Recorded diagnoses were categorized as chronic cervicitis, LSIL, HSIL, or cervical cancer.

### 2.6 Vaginal microecological assessment

Microbiome status was categorized as bacterial vaginosis (BV), trichomonal vaginitis (TV), vulvovaginal candidiasis (VVC), aerobic vaginitis (AV), mixed infection, suppressed flora, or normal controls (NC). Wet mounts examined under 400× magnification assessed cleanliness, *Candida*, and *Trichomonas* morphology. Vaginal pH was measured using a dedicated test kit, while Swiss-stained smears evaluated *lactobacillus* abundance, microbial density, and diversity at 1000× magnification. *Candida* identification relied on visualizing spores or hyphae, *Trichomonas* on flagellar morphology, and cleanliness grading on leukocyte, bacterial, coccal, and epithelial cell quantification.

### 2.7 Statistical analysis

Statistical analyses were performed using R version 4.4.2. Continuous variables with non-normal distributions were summarized as medians and interquartile ranges (IQRs). Age distributions between two groups were compared using the Mann–Whitney U test, whereas comparisons among three or more groups were performed using the Kruskal–Wallis test. Categorical variables were summarized as counts and percentages and compared using Pearson’s χ² test; Fisher’s exact test was used when expected cell counts were small. Genotype-specific prevalence was calculated at the participant level, with each genotype counted no more than once per participant within a given analysis. Participants with multiple-type infections could therefore contribute to more than one genotype category. For comparisons of the 21 HPV genotypes across cervical histopathological or vaginal microecological categories, genotype-level *P* values were adjusted using the Benjamini–Hochberg false discovery rate (FDR) procedure to account for multiple testing. Statistical significance was defined as a two-sided *P* < 0.05 for unadjusted analyses and an FDR-adjusted *P* < 0.05 for genotype-specific multiple comparisons. Bar plots, line plots, violin plots, chord diagrams, and heatmaps were generated using R and Hiplot Pro(https://hiplot.com.cn/), as appropriate.

## 3 Results

### 3.1 Demographic and clinical characteristics

Between January 2020 and December 2024, a total of 73,828 individuals were included in the analysis, comprising 69,771 females (94.50%) and 4,057 males (5.50%). Age distributions differed significantly by sex, with a median age of 41 years (IQR, 34–49) in females and 32 years (IQR, 26–39) in males (Mann–Whitney *U* = 200,921,160.5, *P* < 0.001; rank-biserial *r* = 0.420; **Figure 1A**).

**Figure 1:**
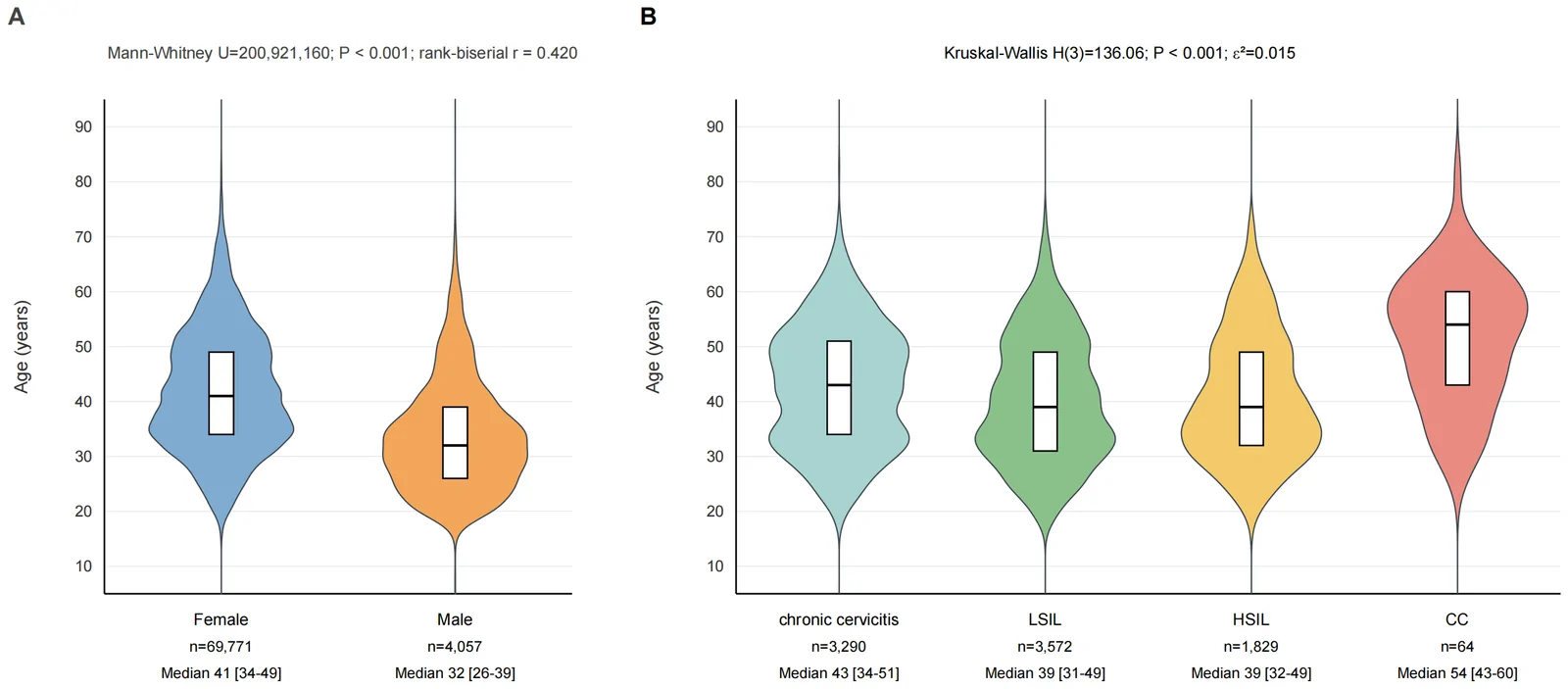
Age distributions according to sex and cervical histopathological category. **(A)** Age distributions in female and male participants. Differences were evaluated using the two-sided Mann–Whitney U test; rank-biserial r is reported as the effect size. **(B)** Age distributions among women with chronic cervicitis, low-grade squamous intraepithelial lesion (LSIL), high-grade squamous intraepithelial lesion (HSIL), and cervical cancer (CC).

Among the 69,771 female participants, 8,755 had both HPV genotyping results and cervical histopathological records and were included in the histopathological analysis. The histopathological categories included chronic cervicitis (n = 3,290, 37.58%), LSIL (n = 3,572, 40.80%), HSIL (n = 1,829, 20.90%), and Cervical cancer (CC; n = 64, 0.73%). Age distributions differed significantly across histopathological categories (Kruskal–Wallis *H*(3) = 136.06, *P* < 0.001; ϵ² = 0.015). Median ages were 43 years (IQR, 34–51) for chronic cervicitis, 39 years (IQR, 31–49) for LSIL, 39 years (IQR, 32–49) for HSIL, and 54 years (IQR, 43–60) for CC. Dunn’s *post hoc* tests with Holm correction showed no significant difference between the LSIL and HSIL groups (adjusted *P* = 0.1516), whereas all other pairwise comparisons were statistically significant (all adjusted *P* < 0.001; **Figure 1B**).

### 3.2 The overall prevalence and characteristics of HPV infection

Among the 73,828 participants,20.83% (15,376/73,828) of participants were tested positive for one or more HPV genotypes. When infections containing at least one genotype from each risk category were considered, HR-HPV was detected in 12,759 individuals (17.28%), IR-HPV in 1,094 (1.48%), and LR-HPV in 3,333 (4.51%). Because individuals with multiple-type infections could harbor genotypes from more than one risk category, these categories were not mutually exclusive. Among HPV-positive individuals, single-type infections accounted for 77.05% (11,847/15,376), double infections for 17.35% (2,667/15,376), and infections involving three or more genotypes for 5.61% (862/15,376). All 21 HPV genotypes targeted by the assay were detected, including 14 HR-HPV genotypes, one IR-HPV genotype, and six LR-HPV genotypes.

HPV16 was the most frequently detected genotype (3,089/73,828, 4.18%), followed by HPV52 (2,620/73,828, 3.55%), HPV58 (1,882/73,828, 2.55%), HPV51 (1,291/73,828, 1.75%), and HPV39 (1,220/73,828, 1.65%). Among LR-HPV genotypes, HPV81 was the most frequently detected (917/73,828, 1.24%), followed by HPV6 (834/73,828, 1.13%), HPV42 (619/73,828, 0.84%), HPV11 (538/73,828, 0.73%), HPV43 (370/73,828, 0.50%), and HPV44 (264/73,828, 0.36%) (**Figure 2A**). Age-specific trends for the predominant HR-HPV genotypes are shown in **Figure 2B**.

**Figure 2:**
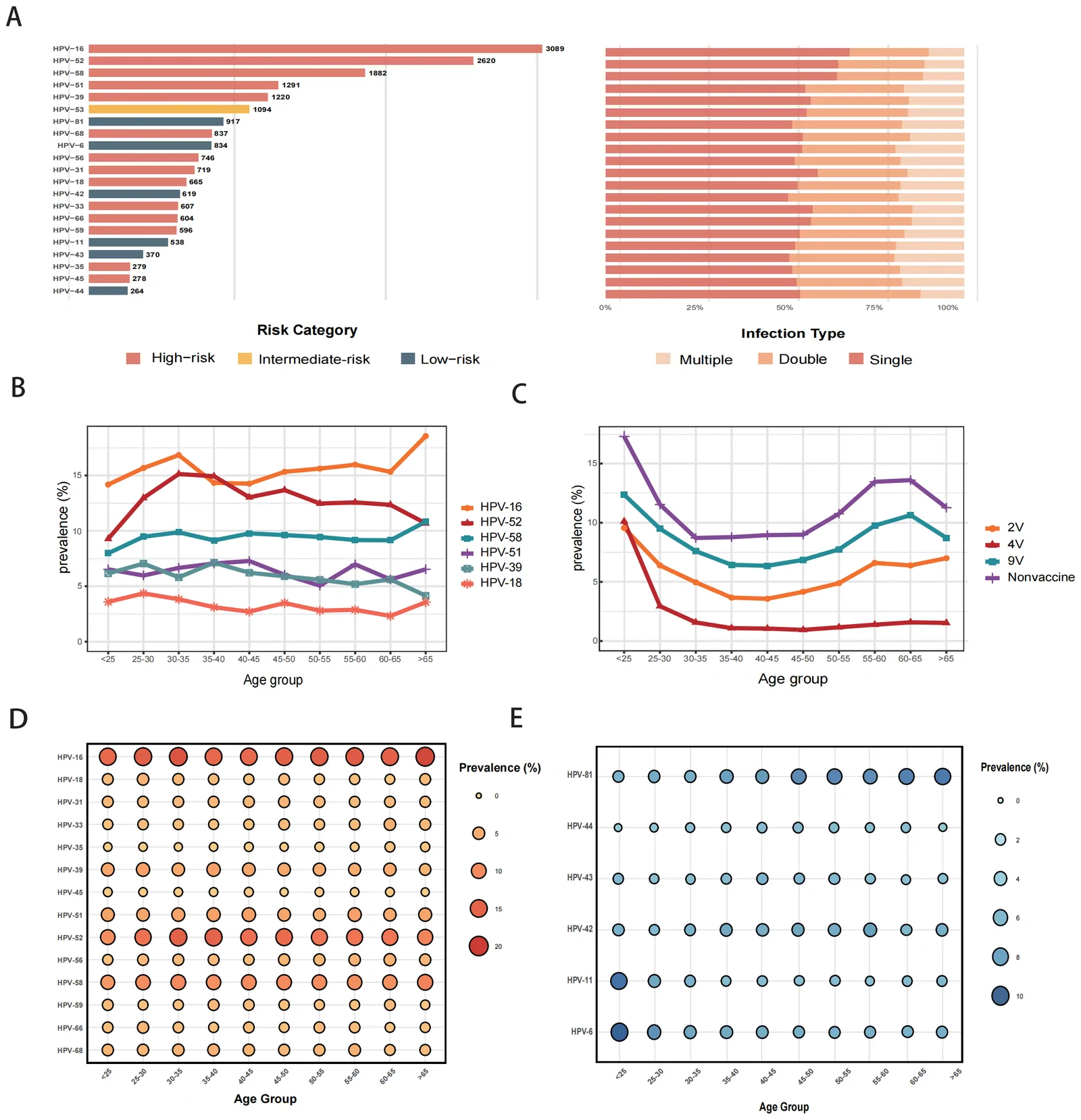
**(A)** HPV genotype burden and infection type. Left: Vertical bars quantify total infections per genotype, stratified by oncogenic risk (high/medium/low, color-coded). Right: Stacked bars delineate proportions of single, dual, and multiple infections within each genotype. **(B)** Longitudinal prevalence of predominant HPV genotypes.Age-cohort trends (<25 to >65 years) are shown for clinically dominant genotypes. **(C)** Differential prevalence of vaccine-relevant genotypes.Age-stratified curves contrast 2V/4V/9V-included genotypes against non-vaccine types.bivalent(2v:HPV16/18);quadrivalent(4v:HPV6/11/16/18);nonavalent(9v:HPV6/11/16/18/31/33/45/52/58). **(D)** High-risk HPV epidemiology by age. Proportional bubble encoding (size/color) maps prevalence variations of oncogenic types (red-orange spectrum) across age deciles. **(E)** Low-risk HPV age distribution. Analogous bubble plot for non-oncogenic types (blue spectrum).

### 3.3 Age-specific patterns of HPV infection

Participants were stratified into ten age groups, ranging from < 25 to > 65 years. As shown in **Table 1**, the prevalence of overall HPV infection, single-genotype infection categories, cumulative vaccine-covered HPV, and non-9V genotypes differed significantly across age groups (all χ² tests *P* < 0.001). The overall HPV positivity rate was 20.83% (15,376/73,828) and showed a distinct bimodal age distribution, with the highest prevalence in participants aged < 25 years (34.44%) and a second peak at 60–65 years (25.90%); the lowest prevalence was observed at 40–45 years (17.45%).Single HR-HPV infections were most prevalent in the 25–30 year group (15.41%), whereas single LR-HPV infections were most frequent in participants aged < 25 years (5.82%). Single IR-HPV infections were generally more common in older age groups and reached their highest prevalence at 60–65 years (1.46%).

**Table 1:** Age-stratified prevalence of HPV infection patterns and cumulative vaccine-covered genotypes.

Variables	<25 (n = 3799)	25-30 (n = 6449)	30-35 (n = 12026)	35-40 (n = 13269)	40-45 (n = 11066)	45-50 (n = 9479)	50-55 (n = 8338)	55-60 (n = 4845)	60-65 (n = 2471)	>65 (n = 2086)	Total (n = 73828)	X^2^	*P-*value
Positive Cases	1306 (34.44)	1617 (25.07)	2348 (19.52)	2329 (17.55)	1931 (17.45)	1733 (18.28)	1754 (21.04)	1244 (25.68)	640 (25.90)	474 (22.72)	15376 (20.83)	821.201	<0.001
Single-HR	544 (14.32)	994 (15.41)	1538 (12.79)	1536 (11.58)	1255 (11.34)	1110(11.71)	1077 (12.92)	702 (14.49)	355 (14.37)	260(12.46)	9371 (12.69)	118.851	<0.001
Single-IR	23 (0.61)	49 (0.76)	82 (0.68)	98 (0.74)	93 (0.84)	71 (0.75)	83 (1.00)	56 (1.16)	36 (1.46)	21(1.01)	612 (0.83)	64.313	<0.001
Single-LR	221 (5.82)	186 (2.88)	254 (2.11)	288 (2.17)	224 (2.02)	232 (2.45)	206 (2.47)	138 (2.85)	63 (2.55)	52 (2.49)	1864 (2.52)	199.491	<0.001
2V	363 (9.56)	412 (6.39)	596 (4.96)	487 (3.67)	395 (3.57)	394 (4.16)	408 (4.89)	320 (6.60)	158 (6.39)	146 (7.00)	3,679 (4.98)	358.673	<0.001
4V	675 (17.77)	577 (8.95)	761 (6.33)	617 (4.65)	502 (4.54)	475 (5.01)	493 (5.91)	376 (7.76)	193 (7.81)	173 (8.29)	4,842 (6.56)	1,063.393	<0.001
9V	968 (25.48)	1,097 (17.01)	1,569 (13.05)	1,405 (10.59)	1,148 (10.37)	1,077 (11.36)	1,083 (12.99)	786 (16.22)	429 (17.36)	325 (15.58)	9,887 (13.39)	839.978	<0.001
Nonvaccine	657 (17.29)	744 (11.54)	1047 (8.71)	1165 (8.78)	990 (8.95)	852 (8.99)	897 (10.76)	652 (13.46)	336 (13.60)	235 (11.27)	7575(10.26)	404.275	<0.001

HPV, human papillomavirus; HR, high-risk HPV; LR, low-risk HPV; IR, Intermediate-Risk HPV; bivalent (2v: HPV16/18); quadrivalent (4v: HPV6/11/16/18); nonavalent (9v: HPV6/11/16/18/31/33/45/52/58).

Cumulative vaccine-covered HPV prevalence also showed marked age-related variation. The overall prevalence of 2V-, 4V-, and 9V-covered HPV was 4.98%, 6.56%, and 13.39%, respectively. All three cumulative vaccine-covered categories showed their highest prevalence in participants aged < 25 years, reaching 9.56% for 2V-covered HPV, 17.77% for 4V-covered HPV, and 25.48% for 9V-covered HPV. Prevalence declined during middle adulthood and increased again in older age groups, with 2V-, 4V-, and 9V-covered HPV reaching 7.00%, 8.29%, and 15.58%, respectively, in participants aged > 65 years. At least one non-9V genotype was detected in 10.26% of the overall cohort, with the highest prevalence observed in participants aged < 25 years (17.29%) (**Figure 2C**).

Age-specific distributions of HR-HPV genotypes are shown in **Figure 2D**. HPV16, HPV52, and HPV58 were among the most frequently detected HR-HPV genotypes at both younger and older age extremes. Among LR-HPV genotypes, HPV6, HPV81, and HPV42 showed relatively high prevalence in the <25 year group (**Figure 2E**).

### 3.4 Overall and distribution of single, double, and multiple HPV infections

Among the 15,376 HPV-positive individuals, 14,636 (95.19%) were female and 740 (4.81%) were male. Single-type infections accounted for 77.05% (11,847/15,376) of HPV-positive cases, whereas double- and multiple-type infections accounted for 17.35% (2,667/15,376) and 5.61% (862/15,376), respectively. The relative distribution of single-, double-, and multiple-type infections did not differ significantly between females and males (χ² = 0.21, *P* = 0.898), whereas infection multiplicity differed significantly across age groups (χ² = 516.77, *P* < 0.001; **Table 2**). Sex-stratified distributions of infection multiplicity across age groups are shown in **Figure 3A**.

**Table 2:** Single and multiple infection status in 15376 people with HPV infection.

Variables	Single infection (n = 11847)	Double infections (n = 2667)	Multiple infections (n = 862)	Total infections (n = 15376)	χ²	*p*-value
Gender, n (%)					0.21	0.898
Man	566 (4.78)	130 (4.87)	44 (5.10)	740 (4.81)		
Woman	11281 (95.22)	2537 (95.13)	818 (94.90)	14636 (95.19)		
Age group, n (%)					516.77	<.001
<25	788 (6.65)	308 (11.55)	210 (24.36)	1306 (8.49)		
25-30	1229 (10.37)	307 (11.51)	81 (9.40)	1617 (10.52)		
30-35	1874 (15.82)	386 (14.47)	88 (10.21)	2348 (15.27)		
35-40	1922 (16.22)	329 (12.34)	78 (9.05)	2329 (15.15)		
40-45	1572 (13.27)	303 (11.36)	56 (6.50)	1931 (12.56)		
45-50	1413 (11.93)	266 (9.97)	54 (6.26)	1733 (11.27)		
50-55	1366 (11.53)	294 (11.02)	94 (10.90)	1754 (11.41)		
55-60	896 (7.56)	246 (9.22)	102 (11.83)	1244 (8.09)		
60-65	454 (3.83)	132 (4.95)	54 (6.26)	640 (4.16)		
>65	333 (2.81)	96 (3.60)	45 (5.22)	474 (3.08)		

**Figure 3:**
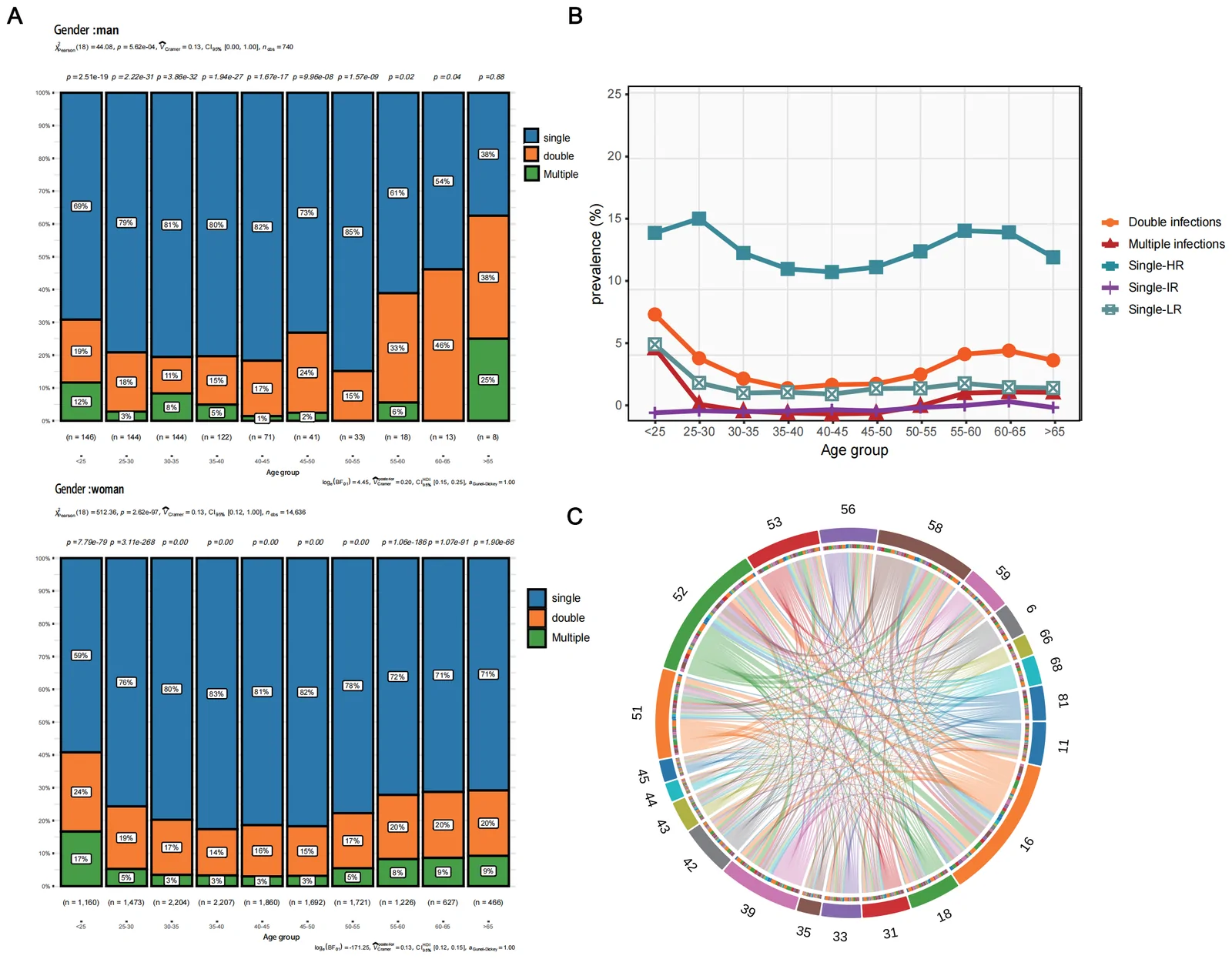
**(A)** gender- and age-stratified distribution of HPV infection types. Clustered bar plots depict percentages of single- (blue), dual- (orange), and multiple-type (green) infections across age groups, separately for females (upper) and males (lower). **(B)** Age-specific prevalence of HPV infection categories. Line graphs illustrate prevalence trends of single high-risk (teal), single Intermediate-risk (purple), single low-risk (cyan), dual (orange), and multiple (red) infections by age cohort. **(C)** Genotype interaction network in mixed infections. Chord diagram visualizes co-occurrence frequencies of HPV genotypes (outer colored segments); ribbon width corresponds to combination prevalence.

Age-specific prevalence showed distinct patterns across infection categories. Single HR-HPV infection remained the predominant category across all age groups, ranging approximately from 11% to 15% and reaching its highest prevalence at 25–30 years (15.41%). Double-type infection was most prevalent in participants aged < 25 years (8.11%), declined during middle adulthood to a minimum of 2.48% at 35–40 years, and increased again in older age groups, reaching 5.34% at 60–65 years. Multiple-type infection showed a similar age-related pattern, with the highest prevalence in participants aged < 25 years (5.53%), the lowest prevalence at 40–45 years (0.51%), and a secondary increase at 60–65 years (2.19%). Single LR-HPV infection was also highest in the < 25-year group (5.82%), whereas single IR-HPV infection remained uncommon across all age groups (**Figure 3B**).

Genotype distributions varied according to infection multiplicity (**Table 3**). Among individuals with single-type infections, HPV16 was the most frequently detected genotype (17.73%), followed by HPV52 (14.32%), HPV58 (10.24%), and HPV51 (6.06%). The same leading genotypes were observed among double-type infections, with HPV16 detected in 25.61%, HPV52 in 23.70%, HPV58 in 16.99%, and HPV51 in 13.35% of cases. Their representation was further increased among individuals with multiple-type infections, in whom HPV16 (35.38%) and HPV52 (33.76%) remained the two most frequently detected genotypes, followed by HPV51 (25.17%) and HPV58 (25.06%). The most frequently observed genotype pairs in co-infections were HPV16/52 (n = 174), HPV16/58 (n = 138), HPV52/58 (n = 133), HPV16/51 (n = 119), and HPV52/53 (n = 104) (**Figure 3C**).

**Table 3:** Distribution of HPV genotypes by infection category.

HPV genotype	Single n (%) [95% CI] N=11,847	Double n (%) [95% CI] N=2,667	Multiple n (%) [95% CI] N=862	Overall n (%) [95% CI] N=15,376
HPV-6	457 (3.86) [3.53-4.22]	218 (8.17) [7.19-9.27]	159 (18.45) [16.00-21.17]	834 (5.42) [5.08-5.79]
HPV-11	284 (2.40) [2.14-2.69]	152 (5.70) [4.88-6.64]	102 (11.83) [9.84-14.16]	538 (3.50) [3.22-3.80]
HPV-16	2,101 (17.73) [17.06-18.43]	683 (25.61) [23.99-27.30]	305 (35.38) [32.26-38.63]	3,089 (20.09) [19.46-20.73]
HPV-18	356 (3.00) [2.71-3.33]	191 (7.16) [6.24-8.20]	118 (13.69) [11.55-16.15]	665 (4.32) [4.01-4.66]
HPV-31	425 (3.59) [3.27-3.94]	180 (6.75) [5.86-7.76]	114 (13.23) [11.13-15.65]	719 (4.68) [4.35-5.02]
HPV-33	350 (2.95) [2.66-3.27]	170 (6.37) [5.51-7.37]	87 (10.09) [8.26-12.28]	607 (3.95) [3.65-4.27]
HPV-35	145 (1.22) [1.04-1.44]	84 (3.15) [2.55-3.88]	50 (5.80) [4.43-7.57]	279 (1.81) [1.62-2.04]
HPV-39	697 (5.88) [5.47-6.32]	335 (12.56) [11.36-13.87]	188 (21.81) [19.18-24.69]	1,220 (7.93) [7.52-8.37]
HPV-42	315 (2.66) [2.38-2.96]	191 (7.16) [6.24-8.20]	113 (13.11) [11.02-15.53]	619 (4.03) [3.73-4.35]
HPV-43	189 (1.60) [1.38-1.84]	109 (4.09) [3.40-4.91]	72 (8.35) [6.69-10.39]	370 (2.41) [2.18-2.66]
HPV-44	143 (1.21) [1.03-1.42]	89 (3.34) [2.72-4.09]	32 (3.71) [2.64-5.19]	264 (1.72) [1.52-1.93]
HPV-45	148 (1.25) [1.06-1.47]	82 (3.07) [2.48-3.80]	48 (5.57) [4.23-7.31]	278 (1.81) [1.61-2.03]
HPV-51	718 (6.06) [5.65-6.50]	356 (13.35) [12.11-14.69]	217 (25.17) [22.39-28.18]	1,291 (8.40) [7.97-8.85]
HPV-52	1,697 (14.32) [13.71-14.97]	632 (23.70) [22.12-25.35]	291 (33.76) [30.68-36.98]	2,620 (17.04) [16.45-17.64]
HPV-53	612 (5.17) [4.78-5.58]	311 (11.66) [10.50-12.93]	171 (19.84) [17.31-22.63]	1,094 (7.11) [6.72-7.53]
HPV-56	393 (3.32) [3.01-3.66]	221 (8.29) [7.30-9.39]	132 (15.31) [13.06-17.87]	746 (4.85) [4.52-5.20]
HPV-58	1,213 (10.24) [9.71-10.80]	453 (16.99) [15.61-18.46]	216 (25.06) [22.28-28.06]	1,882 (12.24) [11.73-12.77]
HPV-59	322 (2.72) [2.44-3.03]	175 (6.56) [5.68-7.57]	99 (11.48) [9.53-13.79]	596 (3.88) [3.58-4.19]
HPV-66	346 (2.92) [2.63-3.24]	170 (6.37) [5.51-7.37]	88 (10.21) [8.36-12.41]	604 (3.93) [3.63-4.25]
HPV-68	460 (3.88) [3.55-4.25]	250 (9.37) [8.33-10.54]	127 (14.73) [12.52-17.26]	837 (5.44) [5.10-5.81]
HPV-81	476 (4.02) [3.68-4.39]	282 (10.57) [9.46-11.80]	159 (18.45) [16.00-21.17]	917 (5.96) [5.60-6.35]

A genotype was counted no more than once per participant record within each column. In double and multiple infections, one participant can contribute to several genotype rows; consequently, genotype percentages across rows sum to more than 100% in those columns. Multiple infection was defined as detection of three or more HPV genotypes. Confidence intervals are two-sided 95% Wilson intervals. CI, confidence interval; HR, high-risk; IR, intermediate-risk; LR, low-risk.

### 3.5 HPV prevalence and genotype distribution among participants with genital and common warts

Retrospective matching of HPV testing records with clinical diagnoses identified 734 participants with documented genital or common warts, including 256 males and 478 females (**Table 4**). Among male participants, 213 had genital warts and 43 had common warts, with HPV positivity rates of 35.21% (75/213) and 18.60% (8/43), respectively. Among female participants, the corresponding positivity rates were 63.56% (293/461) and 29.41% (5/17).

**Table 4:** HPV infection patterns among participants with genital and common warts.

Variables	Man (256)		Woman (478)	
	Genital warts (213)	Common warts (43)	Genital warts (461)	Common warts (17)
Single-HR	4 (1.88)	3 (6.98)	58 (12.58)	2 (11.76)
Single-IR	1 (0.47)	0 (0.00)	2 (0.43)	0 (0.00)
Single-LR	47 (22.07)	4 (9.30)	107 (23.21)	1 (5.88)
Double infections	15 (7.04)	1 (2.33)	67 ( (14.53)	2 (11.76)
Multiple infections	8 (3.76)	0 (0.00)	59 (12.80)	0 (0.00)
Positive Cases	75 (35.21)	8 (18.60)	293 (63.56)	5 (29.41)

Values are n (%), where percentages are calculated using the total number of participants in each sex- and wart-specific subgroup as the denominator.

In participants with genital warts, single LR-HPV infection represented the most common infection category in both males (22.07%) and females (23.21%). HPV6 was the most frequently detected genotype in the genital-wart subgroup, followed by HPV11 and several HR-HPV genotypes, including HPV16, HPV52, HPV51, HPV58, and HPV39 (**Figure 4**). Although HPV6 was detected among participants with common warts, the limited number of cases, particularly among females (n = 17), did not permit reliable assessment of genotype-specific patterns or sex-related differences in this subgroup.

**Figure 4:**
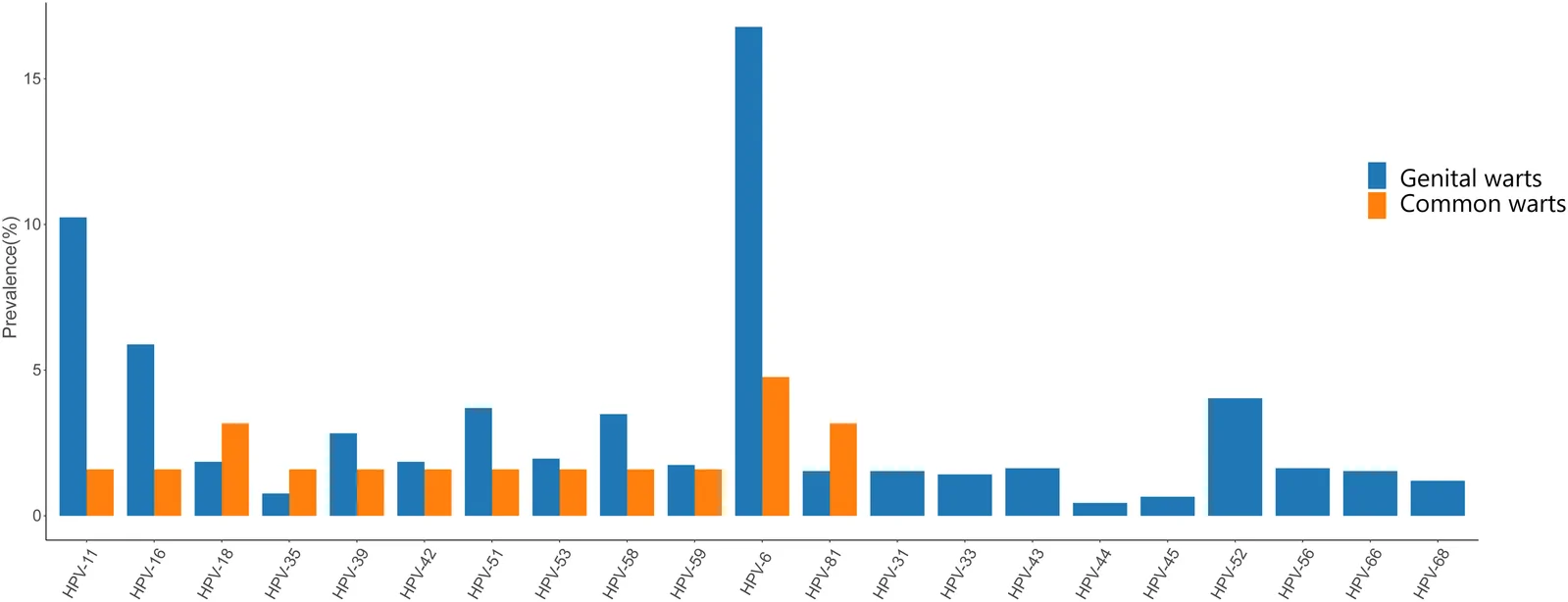
Differential detection frequencies of HPV genotypes by wart. Paired vertical bars illustrate genotype-specific prevalence (%) in genital warts (blue) and common warts (orange).

### 3.6 HPV prevalence and genotype distribution across cervical histopathological categories

Among the 8,755 women included in the histopathological analysis, 4,647 (53.08%) tested positive for at least one HPV genotype. HPV infection patterns differed significantly across cervical histopathological categories. Overall HPV positivity was 38.91% in chronic cervicitis, 61.98% in LSIL, 60.36% in HSIL, and 76.56% in cervical cancer (CC) (χ² = 432.16, *P* < 0.001; **Table 5**). HPV16 was detected in 30.95% of women with HSIL and 46.88% of those with cervical cancer, while HPV18 was detected in 1.86% and 7.81%, respectively. Single HR-HPV infection was the predominant infection category in all groups, accounting for 26.32%, 37.51%, 41.88%, and 57.81% of chronic cervicitis, LSIL, HSIL, and CC cases, respectively (χ² = 171.45, *P* < 0.001). Double-type infections were more frequent in LSIL (12.93%), HSIL (11.97%), and CC (12.50%) than in chronic cervicitis (7.26%), whereas multiple-type infections were most frequent in LSIL (5.52%) and less common in HSIL (3.61%), chronic cervicitis (1.70%), and CC (1.56%). Single IR-HPV and single LR-HPV infections were relatively uncommon across all histopathological categories.

**Table 5:** HPV infection status distribution of cervical lesion status.

Variables	Chronic cervicitis (n = 3290)	LSIL (n = 3572)	HSIL (n = 1829)	CC (n = 64)	Total (n = 8755)	χ²	*p*-value
Single-HR	866 (26.32)	1340 (37.51)	766 (41.88)	37 (57.81)	3009 (34.37)	171.453	<0.001
Single-IR	55 (1.67)	86 (2.41)	15 (0.82)	0 (0.00)	156 (1.78)	19.048	<0.001
Single-LR	64 (1.95)	129 (3.61)	38 (2.08)	3 (4.69)	234 (2.67)	22.281	<0.001
Double infections	239 (7.26)	462 (12.93)	219 (11.97)	8 (12.50)	928 (10.60)	63.048	<0.001
Multiple infections	56 (1.70)	197 (5.52)	66 (3.61)	1 (1.56)	320 (3.66)	71.534	<0.001
Positive Cases	1280(38.91)	2214(61.98)	1104(60.36)	49(76.56)	4647(53.08)	432.161	<0.001

Genotype-specific patient-level prevalence also varied across histopathological categories. HPV16 showed the most pronounced difference, increasing from 9.79% in chronic cervicitis and 13.66% in LSIL to 30.95% in HSIL and 46.88% in CC (FDR-adjusted *P* < 0.001). In contrast, HPV52 was most prevalent in LSIL (12.07%) and was less frequently detected in HSIL (8.42%) and CC (3.12%). HPV58 showed a similar pattern, with prevalence of 9.07% in LSIL and 8.53% in HSIL, compared with 4.69% in CC. HPV18 was detected in 2.95%, 4.54%, 1.86%, and 7.81% of chronic cervicitis, LSIL, HSIL, and CC cases, respectively. Most genotype-specific differences remained statistically significant after Benjamini–Hochberg FDR correction, whereas HPV45 and HPV44 did not show significant variation across pathology groups (**Figure 5A**; **Supplementary Table S1**).

**Figure 5:**
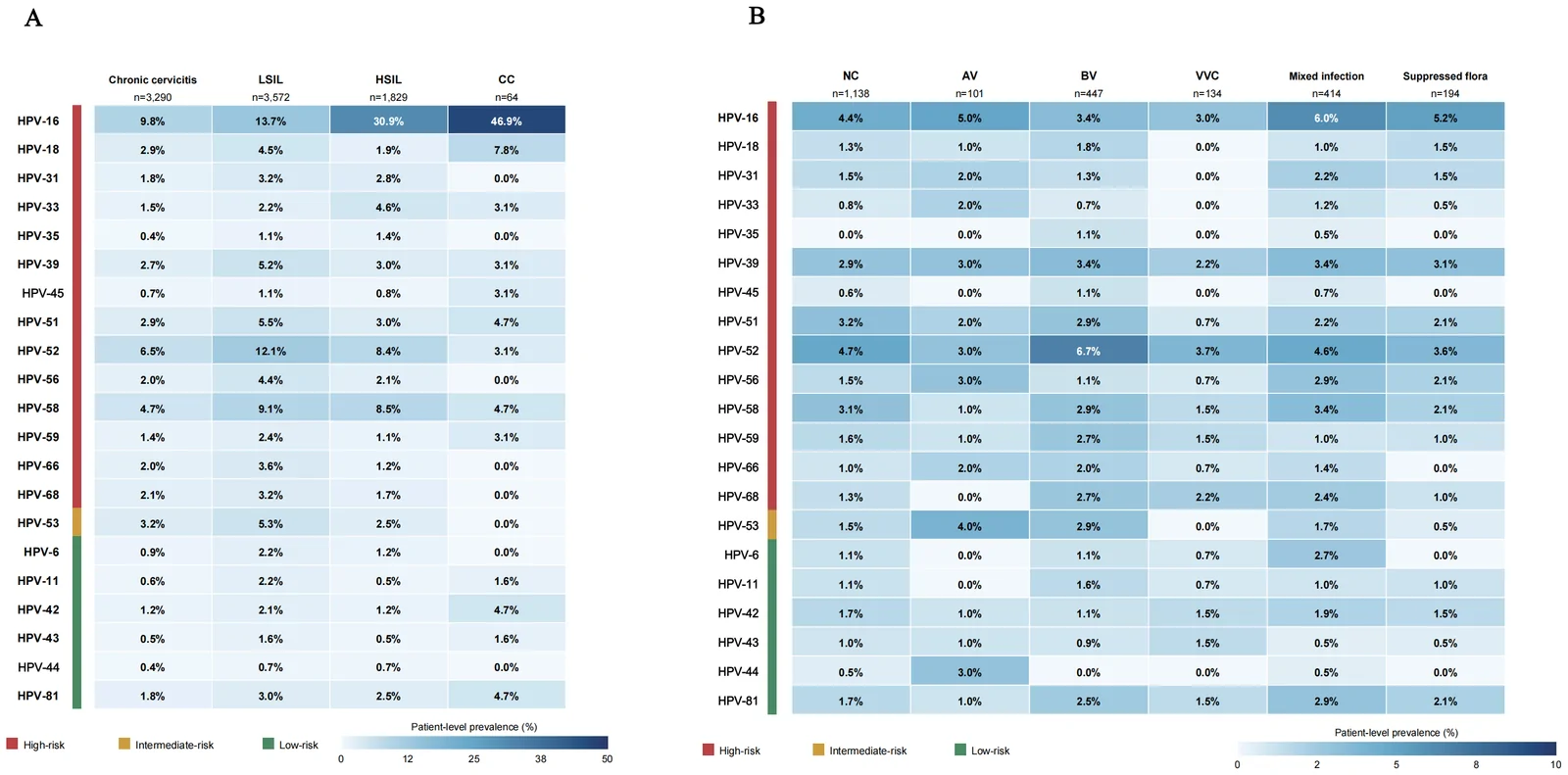
Patient-level prevalence of HPV genotypes across cervical histopathological and vaginal microecological categories. **(A)** Distribution of 21 HPV genotypes across chronic cervicitis, LSIL, HSIL, and cervical cancer. **(B)** Distribution of 21 HPV genotypes across normal flora, AV, BV, VVC, mixed infection, and suppressed flora. Values represent the percentage of all patients in each corresponding group who carried the indicated genotype. Patients with multiple-type infections may contribute to more than one genotype row.

### 3.7 HPV infection patterns across vaginal microecological categories

Among the 2,428 women with paired HPV genotyping and vaginal microecological assessments, 1,138 were classified as normal flora (NC), 101 as aerobic vaginitis (AV), 447 as bacterial vaginosis (BV), 134 as vulvovaginal candidiasis (VVC), 414 as mixed infection, and 194 as suppressed flora. Overall HPV positivity differed significantly across the six microecological categories (χ² = 24.61, *P* < 0.001), with the highest positivity observed in the BV group (32.66%), followed by mixed infection (31.64%), NC (28.56%), AV (27.72%), suppressed flora (22.16%), and VVC (13.43%) (**Table 6**).

**Table 6:** Distribution of HPV infection patterns across vaginal microecological categories.

Variables	Normal flora (n = 1138)	AV (n = 101)	BV (n = 447)	VVC (n = 134)	Mixed infection (n = 414)	Suppressed flora (n = 194)	Total (n = 2428)	χ²	*p*-value
Single-HR	196 (17.22)	16 (15.84)	82 (18.34)	10 (7.46)	70 (16.91)	26 (13.40)	400 (16.47)	10.925	0.053
Single-IR	10 (0.88)	3 (2.97)	11 (2.46)	0 (0.00)	1 (0.24)	0 (0.00)	25 (1.03)	–	0.0043*
Single-LR	50 (4.39)	5 (4.95)	12 (2.68)	2 (1.49)	23 (5.56)	6 (3.09)	98 (4.04)	–	>0.999*
Double infections	50 (4.39)	1 (0.99)	32 (7.16)	3 (2.24)	28 (6.76)	8 (4.12)	122 (5.02)	13.792	0.017
Multiple infections	19 (1.67)	3 (2.97)	9 (2.01)	3 (2.24)	9 (2.17)	3 (1.55)	46 (1.89)	–	>0.999*
HPV-positive	325(28.56)	28(27.72)	146(32.66)	18(13.43)	131(31.64)	43(22.16)	691(28.46)	24.607	<0.001

*Fisher test; χ², Chi-square test; BV, bacterial vaginosis; VVC, vulvovaginal candidiasis; AV, aerobic vaginitis.

Most HPV infection-pattern categories showed only modest variation across vaginal microecological groups. Single HR-HPV infection did not differ significantly among groups (*P* = 0.053). In contrast, single IR-HPV infection showed a significant overall difference (*P* = 0.0043), with relatively higher proportions in AV (2.97%) and BV (2.46%), while double-type infections also varied across groups (χ² = 13.79, *P* = 0.017), with the highest proportions in BV (7.16%) and mixed infection (6.76%). No significant differences were observed for single LR-HPV or multiple-type infections.

At the genotype level, patient-level prevalence showed some numerical variation across vaginal microecological categories, including relatively higher HPV52 prevalence in BV (6.71%) and HPV53 prevalence in AV (3.96%). However, none of the 21 genotype-specific comparisons remained statistically significant after Benjamini–Hochberg false discovery rate correction (all FDR-adjusted *P* > 0.05) (**Figure 5B**; **Supplementary Table S2**). Thus, the observed microecological differences were more evident at the level of overall HPV positivity and selected infection patterns than at the level of individual HPV genotypes.

## 4 Discussion

This large-scale retrospective study of 73,828 individuals provides a comprehensive overview of HPV epidemiology, age-specific infection patterns, cervical histopathology, and vaginal microecological status in a hospital-based population from Northeast China. The overall HPV positivity rate was 20.83%, with a bimodal age distribution characterized by the highest prevalence among participants aged < 25 years (34.44%) and a second peak among those aged 60–65 years (25.90%). HPV16, HPV52, and HPV58 were the most frequently detected genotypes, with participant-level prevalence rates of 4.18%, 3.55%, and 2.55%, respectively. HPV infection patterns differed significantly across cervical histopathological categories, with the highest overall HPV positivity observed in cervical cancer. Notably, HPV16 prevalence increased from 9.79% in chronic cervicitis and 13.66% in LSIL to 30.95% in HSIL and 46.88% in cervical cancer. Vaginal microecological status was also associated with differences in overall HPV positivity and selected infection patterns, although no individual HPV genotype remained significantly associated with microecological category after correction for multiple comparisons.

Geographical variation in HPV infection rates and predominant types represents a key epidemiological feature. This study found a five-year HPV prevalence of 20.83% in Jilin Province, aligning with the reported rate of 21.07% (range: 18.42%–31.94%) across 37 Chinese cities. The overall positivity rate among women was 20.98%, marking a significant decline from our previously reported 34.4% (Hao et al., 2020). This rate is consistent with Beijing (21.0%) but slightly higher than Henan Province (19.7%) (X. Wang et al., 2022). In contrast, there were significant differences compared to the results reported by other cities. Chongqing (30.14%) (S. Li et al., 2024), Chengdu (23.28%) (J. Zhang et al., 2024), and Ordos (26.73%) (Sumeng Wang et al., 2024) had higher HPV positivity rates, while Shanghai (18.81%) (X. Li et al., 2022), Xiamen (18.4%) (Shen et al., 2023), Guangzhou (15.21%) (Qi et al., 2025), and Wuhan (16.87%) (Guo et al., 2024) have lower HPV positivity rates.

The overall HPV positivity rate among men in this study was 18.24%, which is lower than the reported rates for men in Beijing (25.19%) (Shuai Wang et al., 2024) and Huizhou (30.53%) (Wen et al., 2025). While research on male HPV infection remains limited, its clinical significance warrants attention: detection rates are significantly higher in men with urethritis, genital warts, HPV-related skin lesions, and prostatitis compared to healthy controls. Meta-analyses support an association between HPV infection and penile cancer (A. Garolla et al., 2024). Furthermore, semen HPV infection rates are approximately 10% in the general male population but rise to 16% in men with unexplained infertility (Andrea Garolla et al., 2013), suggesting a potential role in male reproductive pathophysiology.

The results of this study show that the most prevalent HPV genotypes identified were 16, 52, 58, 51, and 39. This distribution differs from global prevalence trends (16, 18, 45, 33, 58) but aligns with reports from Henan (X. Wang et al., 2022) and Shandong (Zheng et al., 2023) provinces (both: 16, 52, 58, 53, 39). In other regions like Chongqing (S. Li et al., 2024), Chengdu (J. Zhang et al., 2024), and Beijing (W. Zhang et al., 2023), HPV52 prevalence exceeded that of HPV16.Differences in genotype distributions across regions may partly reflect variations in population age structure, clinical characteristics, HPV testing methods, and cervical screening practices. Socioeconomic conditions may contribute indirectly by affecting healthcare access, screening participation, and vaccine uptake. HPV vaccination may also alter the circulation of vaccine-targeted genotypes over time, particularly in populations with sustained high vaccination coverage. However, a previous study conducted in Jilin Province between 2017 and 2019 reported that HPV16, HPV52, and HPV58 were the most prevalent genotypes among women, with HPV53 and HPV51 also common despite not being covered by the currently available nonavalent HPV vaccine (Hao et al., 2020). This regional pattern is consistent with the genotype distribution in our cohort and suggests that the predominance of HPV16, HPV52, and HPV58 may reflect long-standing regional epidemiology rather than a recent vaccine-associated shift. Although HPV vaccination coverage in Jilin Province remains low, with an estimated first-dose coverage of 7.99% among females aged 9–45 years in 2022 (Chen et al., 2024), individual vaccination histories were unavailable. Therefore, the contribution of vaccination to the observed genotype distribution could not be assessed. The pathophysiological progression from HPV infection to cervical cancer initiates in the basal cells of the cervical transformation zone, where actively proliferating squamo-columnar junction cells are susceptible to HPV. Most infections involve episomal viral DNA cleared by the host immune system within 1–2 years. Persistent infection in a minority of cases can lead to viral DNA integration into the host genome, frequently disrupting the viral E2 gene and resulting in constitutive E6/E7 oncogene expression. These oncoproteins then promote cervical carcinogenesis by disrupting p53- and pRb-mediated cell-cycle regulation, leading to malignant transformation. Previous studies have reported associations between E6/E7 expression and the presence of high-grade cervical lesions. This study observed increasing HPV positivity with cervical lesion severity, reaching 76.56% in cervical cancer tissue. This rate is lower than those reported for Chongqing (80.36%) (S. Li et al., 2024), Guangzhou (84.48%) (Yang et al., 2023), Shandong (90.40%) (L. Zhang et al., 2017), and a mainland China meta-analysis (89.9%) (K. Li et al., 2019). HPV16/18 remain the primary oncogenic types: HPV16 prevalence increased significantly with disease progression (consistent with its established oncogenic potential), while HPV18 ranked second, differing from some regional Chinese studies (Yang et al., 2023; Mai et al., 2021; Zhou et al., 2018). Positivity rates for HPV53 and HPV39 decreased with worsening lesions, possibly reflecting genotype-specific differences in immune clearance efficiency or competitive inhibition. Notably, non-vaccine types HPV51 and HPV81 constituted a significant proportion of cervical cancer cases. Other studies suggest HPV33 and HPV58 are emerging as highly carcinogenic in certain regions (Long et al., 2018), informing future vaccine development strategies. While these observations are consistent with the known biological role of high-risk HPV in cervical carcinogenesis, our study was based on HPV DNA genotyping and did not assess persistent infection or E6/E7 expression. Therefore, the observed differences across histopathological categories cannot directly establish a relationship between viral oncoprotein expression and lesion severity.

Age is an important factor in the progression of cervical lesions, with the median age increasing as the severity of the lesions worsens. Our findings also show that HPV prevalence in Jilin Province follows a double-peak age-specific distribution, consistent with reports from other regions (Guo et al., 2024; W. Zhang et al., 2023; J. Zhang et al., 2024). The first peak occurs during adolescence (< 25 years old), which may be related to the onset of sexual activity and hormonal changes after menarche. The second peak occurs in older age (60–65 years old), which may involve immune aging and hormonal changes during perimenopause. Interestingly, a similar “double-peak” pattern is also observed in males. However, the exact mechanisms underlying this “double-peak” pattern remain unclear. Numerous studies have found that HPV infection peaks are showing trends toward both younger and older ages, necessitating appropriate adjustments to the age range for cervical cancer screening and enhanced management of persistent HPV infection.

A healthy vaginal microbiome is characterized by a dynamic balance dominated by *Lactobacillus* species. Their metabolic products, particularly lactic acid, maintain a low vaginal pH (3.8–4.5), essential for cervical epithelial homeostasis (Kovachev, 2018). Microbiome dysbiosis is implicated in persistent HPV infection (Kyrgiou and Moscicki, 2022; Norenhag et al., 2020), potentially through pathogenic bacterial overgrowth reducing *Lactobacillus* abundance, elevating vaginal pH, prolonging squamous epithelial metaplasia, and promoting dysplasia (Boris and Barbés, 2000). Dysbiosis may also disrupt innate vaginal immune defenses, facilitating abnormal HPV adhesion and epithelial damage (Anahtar et al., 2015). In the present study, overall HPV positivity differed significantly across vaginal microecological categories, with higher positivity observed in bacterial vaginosis and mixed infection and the lowest rate in vulvovaginal candidiasis. Differences were also observed for single intermediate-risk HPV and double-type infections, whereas single high-risk HPV, single low-risk HPV, and multiple-type infections did not vary significantly across groups. At the genotype level, several HPV types showed numerical differences among microecological categories; however, none remained significant after correction for multiple comparisons. These findings suggest that vaginal microecological status may be more closely associated with overall HPV positivity and infection pattern than with a specific HPV genotype. Given the retrospective design and relatively small sample sizes in some microecological subgroups, particularly AV and VVC, these associations should be interpreted cautiously and require confirmation in larger prospective studies.

This study delineates HPV prevalence trends in Jilin Province and their association with cervical lesions, providing valuable local epidemiological data. Several limitations should be acknowledged. First, the single-center, hospital-based retrospective design limits the generalizability of our findings. In particular, the histopathological and vaginal microecological subgroups comprised patients with clinically available examination records rather than participants selected through population-based screening. This clinical selection may have enriched these subgroups for women with cervical abnormalities or vaginal symptoms, potentially influencing HPV positivity rates and limiting their comparability with population-based estimates. Second, information on potential confounding factors, including smoking, sexual behavior, and individual HPV vaccination history, was not systematically available. Although individuals with documented HIV infection were excluded, incomplete HIV records did not allow us to rule out residual confounding. Third, detailed cervical cytological and colposcopic information was unavailable for systematic analysis, limiting further interpretation of the clinical findings. Additionally, longitudinal HPV persistence or cervical lesion outcomes could not be assessed. These limitations should be considered when interpreting the observed associations and warrant further investigation in population-based and prospective studies.

## 5 Conclusions

In summary, this large-scale retrospective study of 73,828 individuals provides a comprehensive characterization of HPV epidemiology, age-specific infection patterns, cervical histopathological findings, and vaginal microecological status in a hospital-based population from Northeast China. The overall HPV positivity rate was 20.83%, with HPV16, HPV52, and HPV58 being the most frequently detected genotypes, with participant-level prevalence rates of 4.18%, 3.55%, and 2.55%, respectively. HPV infection patterns varied markedly across age groups and cervical histopathological categories, with HPV16 showing particularly high prevalence in HSIL and cervical cancer. Vaginal microecological status was associated with overall HPV positivity and selected infection patterns, although no individual HPV genotype remained significantly associated with microecological category after correction for multiple comparisons. These findings provide regionally relevant epidemiological evidence that may help refine age- and genotype-informed cervical cancer prevention and screening strategies in Northeast China.

## Data Availability

The original contributions presented in the study are included in the article/**Supplementary Material**, further inquiries can be directed to the corresponding authors.

## References

[B1] Anahtar M. N. Byrne E. H. Doherty K. E. Bowman B. A. Yamamoto H. S. Soumillon M. . (2015). Cervicovaginal bacteria are a major modulator of host inflammatory responses in the female genital tract. Immunity 42, 965–976. doi: 10.1016/j.immuni.2015.04.019 25992865 PMC4461369

[B2] Bautista J. Cortiñas Sardi P. Paguay-Caisabanda I. Gancino-Guevara K. López-Cortés A. (2026). Integrating microbiome insights into cervical cancer. Front. Med. 13, 1766052. doi: 10.3389/fmed.2026.1766052 41846893 PMC12989618

[B3] Boris S. Barbés C. (2000). Role played by lactobacilli in controlling the population of vaginal pathogens. Microbes Infection 2, 543–546. doi: 10.1016/s1286-4579(00)00313-0 10865199

[B4] Bray F. Laversanne M. Sung H. Ferlay J. Siegel R. L. Soerjomataram I. . (2024). Global cancer statistics 2022: GLOBOCAN estimates of incidence and mortality worldwide for 36 cancers in 185 countries. CA: A. Cancer J. For. Clin. 74, 229–263. doi: 10.3322/caac.21834 38572751

[B5] Chaturvedi A. K. Engels E. A. Pfeiffer R. M. Wentzensen N. Anderson W. F. Rosenberg P. S. . (2011). Human papillomavirus and rising oropharyngeal cancer incidence in the United States. J. Clin. Oncol. Off. J. Am. Soc. Clin. Oncol. 29, 4294–4301. doi: 10.1200/JCO.2011.36.4596 21969503 PMC3221528

[B6] Chen J. Zhang Z. Pan W. Song Y. Zheng L. Li L. . (2024). Estimated human papillomavirus vaccine coverage among females 9–45 years of age — China 2017–2022. China CDC Weekly 6, 413–417. doi: 10.46234/ccdcw2024.080 38854753 PMC11153868

[B7] Cheng Z. Zhao Y. Xu T. Wu L. Zhao H. Li J. (2025). Epidemiological characteristics and genotype distribution of human papillomavirus infection in Yangpu District, Shanghai 2020-2024. Virol. J. 22, 225. doi: 10.1186/s12985-025-02855-y 40624568 PMC12232720

[B8] China, National Health Commission Of The People’s Republic Of (2022). National guidelines for diagnosis and treatment of cervical cancer 2022 in China (English version). Chin. J. Cancer Res. = Chung-Kuo Yen Cheng Yen Chiu 34, 256–269. doi: 10.21147/j.issn.1000-9604.2022.03.06 35873886 PMC9273580

[B9] Gao D. Wang X. Juan J. Bi H. Li J. Shi Y. . (2026). Prevalence and genotype distribution of cervical human papillomaviruses infection across cytology: A nationwide, cross-sectional study of over 1 million Chinese women from 2020 to 2024. J. Infection 93, 106773. doi: 10.1016/j.jinf.2026.106773 42217739

[B10] Garolla A. Graziani A. Grande G. Ortolani C. Ferlin A. (2024). HPV-related diseases in male patients: An underestimated conundrum. J. Endocrinol. Invest. 47, 261–274. doi: 10.1007/s40618-023-02192-3 37770654 PMC10859347

[B11] Garolla A. Pizzol D. Bertoldo A. De Toni L. Barzon L. Foresta C. (2013). Association, prevalence, and clearance of human papillomavirus and antisperm antibodies in infected semen samples from infertile patients. Fertility Sterility 99, 125–131.e2. doi: 10.1016/j.fertnstert.2012.09.006 23043686

[B12] Guo W. Hu Z. Yan J. Shen X. Meng Q. Wu H. . (2024). Epidemiological study of human papillomavirus infection in 105,679 women in Wuhan, China. BMC Infect. Dis. 24, 1111. doi: 10.1186/s12879-024-10011-0 39375610 PMC11457396

[B13] Han S. Lin M. Liu M. Wu S. Guo P. Guo J. . (2025). Prevalence, trends, and geographic distribution of human papillomavirus infection in Chinese women: A summative analysis of 2,728,321 cases. BMC Med. 23, 158. doi: 10.1186/s12916-025-03975-6 40082952 PMC11907810

[B14] Hao S. Wang C. Liu S. He J. Jiang Y. (2020). HPV genotypic spectrum in Jilin Province, China, where non-vaccine-covered HPV53 and 51 are prevalent, exhibits a bimodal age-specific pattern. PloS One 15, e0230640. doi: 10.1371/journal.pone.0230640 32208459 PMC7313545

[B15] Höhn A. K. Brambs C. E. Hiller G. G. R. May D. Schmoeckel E. Horn L.-C. (2021). 2020 WHO classification of female genital tumors. Geburtshilfe Und Frauenheilkunde 81, 1145–1153. doi: 10.1055/a-1545-4279 34629493 PMC8494521

[B16] Houvessou G. M. Alfane N. W. A. Mahoche M. (2026). Dynamic, transition and variation of cervicovaginal microbiome and HPV infection and cervical dysplasia and cancer: A systematic review. Infect. Agents Cancer 21, 64. doi: 10.1186/s13027-026-00777-0 42415156 PMC13628985

[B17] Huiyun J. Jie D. Huan W. Yuebo Y. Xiaomao L. (2023). Prevalence and characteristics of cervical human papillomavirus genotypes and cervical lesions among 58630 women from Guangzhou, China. J. Infection Public Health 16, 1531–1536. doi: 10.1016/j.jiph.2023.07.013 37562080

[B18] Jemal A. Simard E. P. Dorell C. Noone A. M. Markowitz L. E. Kohler B. . (2013). Annual report to the nation on the status of cancer 1975-2009, featuring the burden and trends in human papillomavirus(HPV)-associated cancers and HPV vaccination coverage levels. J. Natl. Cancer Institute 105, 175–201. doi: 10.1093/jnci/djs491 23297039 PMC3565628

[B19] Kovachev S. (2018). Defence factors of vaginal lactobacilli. Crit. Rev. Microbiol. 44, 31–39. doi: 10.1080/1040841X.2017.1306688 28418713

[B20] Kyrgiou M. Moscicki A.-B. (2022). Vaginal microbiome and cervical cancer. Semin. Cancer Biol. 86, 189–198. doi: 10.1016/j.semcancer.2022.03.005 35276341

[B21] Li S. He X. Li S. Su Y. Wang X. Li C. (2024). The prevalence of HPV in Chongqing, China from 2017 to 2022: A retrospective cohort study. Sci. Rep. 14, 23973. doi: 10.1038/s41598-024-74588-x 39397097 PMC11471860

[B22] Li K. Li Q. Song L. Wang D. Yin R. (2019). The distribution and prevalence of human papillomavirus in women in mainland China. Cancer 125, 1030–1037. doi: 10.1002/cncr.32003 30748006

[B23] Li X. Xiang F. Dai J. Zhang T. Chen Z. Zhang M. . (2022). Prevalence of cervicovaginal human papillomavirus infection and genotype distribution in Shanghai, China. Virol. J. 19, 146. doi: 10.1186/s12985-022-01879-y 36096810 PMC9465878

[B24] Long W. Yang Z. Li X. Chen M. Liu J. Zhang Y. . (2018). HPV-16, HPV-58, and HPV-33 are the most carcinogenic HPV genotypes in Southwestern China and their viral loads are associated with severity of premalignant lesions in the cervix. Virol. J. 15, 94. doi: 10.1186/s12985-018-1003-x 29801461 PMC5970451

[B25] Mai Q. Yang X. Cheng H. Wu G. Wu Z. (2021). Prevalence and genotype distribution of human papillomavirus among women with cervical lesions in Shenzhen City, China. Hum. Vaccines Immunotherapeutics 17, 965–971. doi: 10.1080/21645515.2020.1805993 32961080 PMC8018457

[B26] Melnikow J. Henderson J. T. Burda B. U. Senger C. A. Durbin S. Weyrich M. S. (2018). Screening for cervical cancer with high-risk human papillomavirus testing: Updated evidence report and systematic review for the US Preventive Services Task Force. JAMA 320, 687–705. doi: 10.1001/jama.2018.10400 30140883 PMC13387487

[B27] Norenhag J. Du J. Olovsson M. Verstraelen H. Engstrand L. Brusselaers N. (2020). The vaginal microbiota, human papillomavirus and cervical dysplasia: A systematic review and network meta-analysis. BJOG: An. Int. J. Obstetrics Gynaecology 127, 171–180. doi: 10.1111/1471-0528.15854 31237400

[B28] Qi S. Tang X. Ye N. Huang W. Wang Y. Li X. (2025). Epidemiological characteristics of human papillomavirus in Guangzhou, China-a retrospective analysis of 76,898 cases. BMC Public Health 25, 2061. doi: 10.1186/s12889-025-23264-4 40462017 PMC12131638

[B29] Schiffman M. Castle P. E. Jeronimo J. Rodriguez A. C. Wacholder S. (2007). Human papillomavirus and cervical cancer. Lancet (London England) 370, 890–907. doi: 10.1016/S0140-6736(07)61416-0 17826171

[B30] Shen Y. Huang Y. Wang W. Zhang J. Chen X. Zhang L. . (2023). Prevalence and genotype distribution of HPV infection among women in Xiamen, China. Front. Microbiol. 14, 1130226. doi: 10.3389/fmicb.2023.1130226 37323886 PMC10265632

[B31] Shi W. Zhu H. Yuan L. Chen X. Huang X. Wang K. . (2022). Vaginal microbiota and HPV clearance: A longitudinal study. Front. Oncol. 12, 955150. doi: 10.3389/fonc.2022.955150 36353544 PMC9639776

[B32] Wang J. Liu X. Liu F. Liu J. Zhao Y. Ma C. . (2026). Associations between vaginal microecological status and high-risk human papillomavirus positivity in women from eastern China: A retrospective cross-sectional study. Front. Med. 13, 1890816. doi: 10.3389/fmed.2026.1890816 42500543 PMC13395778

[B33] Wang S. Liu S. Tan S. Yin J. Li Y. Zhao F. . (2024a). Characteristics of human papillomavirus prevalence and infection patterns among women aged 25-64 according to age groups and cytology results in Ordos City, China. Virol. J. 21, 12. doi: 10.1186/s12985-023-02240-7 38191450 PMC10775550

[B34] Wang S. Ma Q.-Y. Du J. Wei T.-T. Zhang W.-X. Wang P. . (2024b). Detecting and genotyping high-risk human papillomavirus among male patients during 2015-2023 in Beijing, China. Emerging Microbes Infections 13, 2313848. doi: 10.1080/22221751.2024.2313848 38318858 PMC10866056

[B35] Wang X. Song Y. Wei X. Wang G. Sun R. Wang M. . (2022). Prevalence and distribution of human papillomavirus genotypes among women attending gynecology clinics in Northern Henan Province of China. Virol. J. 19, 6. doi: 10.1186/s12985-021-01732-8 34991648 PMC8733907

[B36] Wang D. Yan X. Yang L. Zhang L. (2024). Prevalence and genotype distribution of human papillomavirus infection among women in Wuhan, China: A retrospective study. Infection Drug Resistance 17, 3677–3688. doi: 10.2147/IDR.S471176 39205797 PMC11352622

[B37] Wei F. Georges D. Man I. Baussano I. Clifford G. M. (2024). Causal attribution of human papillomavirus genotypes to invasive cervical cancer worldwide: A systematic analysis of the global literature. Lancet (London England) 404, 435–444. doi: 10.1016/S0140-6736(24)01097-3 39097395

[B38] Wen C. Yang X. Wu X. (2025). HPV infection incidence and genotype distribution among male patients visiting outpatient departments in Huizhou from 2014 to 2023. Virol. J. 22, 105. doi: 10.1186/s12985-025-02726-6 40251597 PMC12007251

[B39] Wirtz C. Mohamed Y. Engel D. Sidibe A. Holloway M. Bloem P. . (2022). Integrating HPV vaccination programs with enhanced cervical cancer screening and treatment, a systematic review. Vaccine 40, A116–A123. doi: 10.1016/j.vaccine.2021.11.013 34863615

[B40] Yan M. Yang Y. Zhao Y. Ma L. Yang Y. Zheng R. (2026). Prevalence and genotype distribution of high-risk human papillomavirus infection among Chinese women aged 35-64: A national screening population-based study. J. Infection 92, 106697. doi: 10.1016/j.jinf.2026.106697 41672253

[B41] Yang X. Li Y. Tang Y. Li Z. Wang S. Luo X. . (2023). Cervical HPV infection in Guangzhou, China: An epidemiological study of 198,111 women from 2015 to 2021. Emerging Microbes Infections 12, e2176009. doi: 10.1080/22221751.2023.2176009 36744409 PMC9936994

[B42] Zhang L. Bi Q. Deng H. Xu J. Chen J. Zhang M. . (2017). Human papillomavirus infections among women with cervical lesions and cervical cancer in eastern China: Genotype-specific prevalence and attribution. BMC Infect. Dis. 17, 107. doi: 10.1186/s12879-017-2223-1 28143439 PMC5282745

[B43] Zhang W. Guo N. Li B. . (2023). Prevalence and genotype distribution of human papillomavirus infections in Beijing, China between 2016 and 2020. Virol. J. 20, 11. doi: 10.1186/s12985-023-01959-7 36653807 PMC9847084

[B44] Zhang J. Zha T. Wang X. He W. (2024). Prevalence and genotype distribution of HPV infections among women in Chengdu,China. Virol. J. 21, 52. doi: 10.1186/s12985-024-02317-x 38429823 PMC10908056

[B45] Zheng L.-L. Chen S.-F. Yang F. Wang W.-H. Xu C. Zheng L.-Y. (2023). High-risk HPV prevalence and genotype distribution among women in Liaocheng, Shandong Province, China from 2016 to 2022. Front. Public Health 11, 1145396. doi: 10.3389/fpubh.2023.1145396 37064671 PMC10098111

[B46] Zhou H.-L. Zhang W. Zhang C.-J. . (2018). Prevalence and distribution of human papillomavirus genotypes in Chinese women between 1991 and 2016: A systematic review. J. Infection 76, 522–528. doi: 10.1016/j.jinf.2018.02.008 29477803

[B47] Žukienė G. Narutytė R. Rudaitis V. (2025). Association between vaginal microbiota and cervical dysplasia due to persistent human papillomavirus infection: A systematic review of evidence from shotgun metagenomic sequencing studies. Int. J. Mol. Sci. 26, 4258. doi: 10.3390/ijms26094258 40362493 PMC12072622

