# Supplementary material for: Age-specific HPV genotype distribution and associations with cervical lesions and vaginal microecological status in Northeast China: a retrospective study of 73,828 individuals
Source: Front Cell Infect Microbiol. 2026 Oct 30;16:1993128. doi: 10.3389/fcimb.2026.1993128 (PMC13759393; doi:10.3389/fcimb.2026.1993128)
Supplement: Supplementary file 1 [file Table1.docx]

**Supplementary**

**Table S1. Patient-level prevalence of HPV genotypes across cervical histopathological categories**

| **HPV genotype** | **Chronic cervicitis n (%) N=3,290** | **LSIL n (%) N=3,572** | **HSIL n (%) N=1,829** | **CC n (%) N=64** | **P value** | **FDR-adjusted P** |
| --- | --- | --- | --- | --- | --- | --- |
| HPV-16 | 322 (9.79) | 488 (13.66) | 566 (30.95) | 30 (46.88) | <0.001 | <0.001 |
| HPV-18 | 97 (2.95) | 162 (4.54) | 34 (1.86) | 5 (7.81) | <0.001 | <0.001 |
| HPV-31 | 59 (1.79) | 115 (3.22) | 52 (2.84) | 0 (0.00) | <0.001 | 0.001 |
| HPV-33 | 49 (1.49) | 79 (2.21) | 84 (4.59) | 2 (3.12) | <0.001 | <0.001 |
| HPV-35 | 13 (0.40) | 39 (1.09) | 26 (1.42) | 0 (0.00) | <0.001 | <0.001 |
| HPV-39 | 88 (2.67) | 186 (5.21) | 54 (2.95) | 2 (3.12) | <0.001 | <0.001 |
| HPV-45 | 24 (0.73) | 39 (1.09) | 14 (0.77) | 2 (3.12) | 0.090 | 0.094 |
| HPV-51 | 97 (2.95) | 196 (5.49) | 55 (3.01) | 3 (4.69) | <0.001 | <0.001 |
| HPV-52 | 214 (6.50) | 431 (12.07) | 154 (8.42) | 2 (3.12) | <0.001 | <0.001 |
| HPV-56 | 65 (1.98) | 156 (4.37) | 39 (2.13) | 0 (0.00) | <0.001 | <0.001 |
| HPV-58 | 156 (4.74) | 324 (9.07) | 156 (8.53) | 3 (4.69) | <0.001 | <0.001 |
| HPV-59 | 47 (1.43) | 84 (2.35) | 21 (1.15) | 2 (3.12) | 0.002 | 0.003 |
| HPV-66 | 65 (1.98) | 129 (3.61) | 22 (1.20) | 0 (0.00) | <0.001 | <0.001 |
| HPV-68 | 70 (2.13) | 113 (3.16) | 32 (1.75) | 0 (0.00) | 0.003 | 0.004 |
| HPV-53 | 104 (3.16) | 189 (5.29) | 45 (2.46) | 0 (0.00) | <0.001 | <0.001 |
| HPV-6 | 29 (0.88) | 77 (2.16) | 22 (1.20) | 0 (0.00) | <0.001 | <0.001 |
| HPV-11 | 19 (0.58) | 79 (2.21) | 10 (0.55) | 1 (1.56) | <0.001 | <0.001 |
| HPV-42 | 38 (1.16) | 76 (2.13) | 22 (1.20) | 3 (4.69) | 0.001 | 0.001 |
| HPV-43 | 17 (0.52) | 58 (1.62) | 10 (0.55) | 1 (1.56) | <0.001 | <0.001 |
| HPV-44 | 12 (0.36) | 25 (0.70) | 12 (0.66) | 0 (0.00) | 0.238 | 0.238 |
| HPV-81 | 60 (1.82) | 107 (3.00) | 46 (2.52) | 3 (4.69) | 0.007 | 0.008 |

Notes: Percentages are patient-level prevalence estimates: the number of patients carrying the indicated genotype divided by all patients in the corresponding pathology group. A genotype was counted no more than once per patient record. Patients with multiple-type infections may contribute to multiple genotype rows; therefore, rows are not mutually exclusive and column percentages may sum to more than 100%. Pearson’s chi-square test was used when all expected cell counts were ≥5; otherwise, the Fisher–Freeman–Halton test with Monte Carlo estimation was used. P values were adjusted across all 21 genotype comparisons using the Benjamini–Hochberg false discovery rate procedure. CC, cervical cancer; FDR, false discovery rate; HR, high-risk; IR, intermediate-risk; LR, low-risk; LSIL, low-grade squamous intraepithelial lesion; HSIL, high-grade squamous intraepithelial lesion. High-risk genotypes: HPV-16, -18, -31, -33, -35, -39, -45, -51, -52, -56, -58, -59, -66, and -68; intermediate-risk: HPV-53; low-risk: HPV-6, -11, -42, -43, -44, and -81.

**Table S2. Patient-level prevalence of HPV genotypes across vaginal microecological categories**

| **HPV genotype** | **NC n (%) N=1,138** | **AV n (%) N=101** | **BV n (%) N=447** | **VVC n (%) N=134** | **Mixed infection n (%) N=414** | **Suppressed flora n (%) N=194** | **P value** | **FDR-adjusted P** |
| --- | --- | --- | --- | --- | --- | --- | --- | --- |
| HPV-16 | 50 (4.39) | 5 (4.95) | 15 (3.36) | 4 (2.99) | 25 (6.04) | 10 (5.15) | 0.465 | 0.937 |
| HPV-18 | 15 (1.32) | 1 (0.99) | 8 (1.79) | 0 (0.00) | 4 (0.97) | 3 (1.55) | 0.714 | 0.937 |
| HPV-31 | 17 (1.49) | 2 (1.98) | 6 (1.34) | 0 (0.00) | 9 (2.17) | 3 (1.55) | 0.594 | 0.937 |
| HPV-33 | 9 (0.79) | 2 (1.98) | 3 (0.67) | 0 (0.00) | 5 (1.21) | 1 (0.52) | 0.566 | 0.937 |
| HPV-35 | 0 (0.00) | 0 (0.00) | 5 (1.12) | 0 (0.00) | 2 (0.48) | 0 (0.00) | 0.012 | 0.254 |
| HPV-39 | 33 (2.90) | 3 (2.97) | 15 (3.36) | 3 (2.24) | 14 (3.38) | 6 (3.09) | 0.985 | 0.985 |
| HPV-45 | 7 (0.62) | 0 (0.00) | 5 (1.12) | 0 (0.00) | 3 (0.72) | 0 (0.00) | 0.678 | 0.937 |
| HPV-51 | 36 (3.16) | 2 (1.98) | 13 (2.91) | 1 (0.75) | 9 (2.17) | 4 (2.06) | 0.655 | 0.937 |
| HPV-52 | 54 (4.75) | 3 (2.97) | 30 (6.71) | 5 (3.73) | 19 (4.59) | 7 (3.61) | 0.481 | 0.937 |
| HPV-56 | 17 (1.49) | 3 (2.97) | 5 (1.12) | 1 (0.75) | 12 (2.90) | 4 (2.06) | 0.247 | 0.741 |
| HPV-58 | 35 (3.08) | 1 (0.99) | 13 (2.91) | 2 (1.49) | 14 (3.38) | 4 (2.06) | 0.779 | 0.957 |
| HPV-59 | 18 (1.58) | 1 (0.99) | 12 (2.68) | 2 (1.49) | 4 (0.97) | 2 (1.03) | 0.498 | 0.937 |
| HPV-66 | 11 (0.97) | 2 (1.98) | 9 (2.01) | 1 (0.75) | 6 (1.45) | 0 (0.00) | 0.216 | 0.741 |
| HPV-68 | 15 (1.32) | 0 (0.00) | 12 (2.68) | 3 (2.24) | 10 (2.42) | 2 (1.03) | 0.208 | 0.741 |
| HPV-53 | 17 (1.49) | 4 (3.96) | 13 (2.91) | 0 (0.00) | 7 (1.69) | 1 (0.52) | 0.052 | 0.367 |
| HPV-6 | 13 (1.14) | 0 (0.00) | 5 (1.12) | 1 (0.75) | 11 (2.66) | 0 (0.00) | 0.094 | 0.493 |
| HPV-11 | 13 (1.14) | 0 (0.00) | 7 (1.57) | 1 (0.75) | 4 (0.97) | 2 (1.03) | 0.927 | 0.985 |
| HPV-42 | 19 (1.67) | 1 (0.99) | 5 (1.12) | 2 (1.49) | 8 (1.93) | 3 (1.55) | 0.953 | 0.985 |
| HPV-43 | 11 (0.97) | 1 (0.99) | 4 (0.89) | 2 (1.49) | 2 (0.48) | 1 (0.52) | 0.820 | 0.957 |
| HPV-44 | 6 (0.53) | 3 (2.97) | 0 (0.00) | 0 (0.00) | 2 (0.48) | 0 (0.00) | 0.031 | 0.325 |
| HPV-81 | 19 (1.67) | 1 (0.99) | 11 (2.46) | 2 (1.49) | 12 (2.90) | 4 (2.06) | 0.664 | 0.937 |

Notes: Percentages are patient-level prevalence estimates: the number of patients carrying the indicated genotype divided by all patients in the corresponding vaginal microecological group. A genotype was counted no more than once per patient record. Patients with multiple-type infections may contribute to multiple genotype rows; therefore, rows are not mutually exclusive and column percentages may sum to more than 100%. Pearson’s chi-square test was used when all expected cell counts were ≥5; otherwise, the Fisher–Freeman–Halton test with Monte Carlo estimation was used. P values were adjusted across all 21 genotype comparisons using the Benjamini–Hochberg false discovery rate procedure. AV, aerobic vaginitis; BV, bacterial vaginosis; FDR, false discovery rate; NC, normal control; VVC, vulvovaginal candidiasis. High-risk genotypes: HPV-16, -18, -31, -33, -35, -39, -45, -51, -52, -56, -58, -59, -66, and -68; intermediate-risk: HPV-53; low-risk: HPV-6, -11, -42, -43, -44, and -81.
